# Application of nanoarchitectonics in moist-electric generation

**DOI:** 10.3762/bjnano.13.99

**Published:** 2022-10-25

**Authors:** Jia-Cheng Feng, Hong Xia

**Affiliations:** 1 State Key Laboratory on Integrated Optoelectronics, College of Electronic Science and Engineering, Jilin University, 2699 Qianjin Street, Changchun130012, China

**Keywords:** electric double layer, energy, moist-electric generators, nanoarchitectonics

## Abstract

The consumption of energy is an important resource that cannot be ignored in modern society. Non-renewable forms of energy, such as coal, natural gas, and oil, have always been important strategic resources and are always facing a crisis of shortage. Therefore, there is an urgent need for green renewable forms of energy. As an emerging green energy source, the moist-electric generator (MEG) has been studied in recent years and may become an energy source that can be utilized in daily life. Along with the advancement of technological means, nanoarchitectonics play an important role in MEG devices. This review aims to provide a comprehensive summary of the fundamentals of the MEG from the perspective of different material classifications and to provide guidance for future work in the field of MEGs. The effects of various parameters and structural designs on the output power, recent important literature and works, the mechanism of liquid–solid interactions at the nanoscale, and the application status and further potential of MEG devices are discussed in this review. It is expected that this review may provide valuable knowledge for future MEG research.

## Review

### 1 Introduction

The use of nanoarchitectonics concept in functional devices has been a hot research topic in recent years, and nanoarchitectonics has a significant impact on the improvement of mechanical structural strength, electrical conductivity, and optoelectronic properties [1–3]. The high porosity of nanostructured materials has a unique application, together with a rapid development in recent years, in the emerging field of moist-electric generators (MEGs), which are primarily based on the electrokinetic effect. The earliest quantitative experiments of the electrokinetic effect were mentioned in 1852 by Gustav Wiedemann [4]. He investigated electro-osmosis in tubes and provided a qualitative explanation of the mechanism. In 1861, Georg Quincke measured a potential difference between the two ends of the channels when water flowed in pipe channels, which implies that the streaming potential may be converted to electric power [5–6]. This viewpoint was confirmed by many experiments later, such as the electrical signal generated by the flow of water through single-walled carbon nanotubes [7], carbon nanosheets [8], and nanoparticles [9]. Regarding the principle of this phenomenon, the common explanation is that charge transfer occurs when a liquid is in contact with a solid with a surface charge. This interaction is mainly dominated by the electric double layer (EDL), which consists of a layer of ions (Stern layer) that is tightly adsorbed to the charged surface and a layer of counter ions (diffusion layer) that is attracted to the surface charges. When the liquid moves in the microchannel, it will drag the diffusion layer ions to form a flowing current, thus creating a potential difference, namely the flowing potential between the two ends of the channel. In nanochannels, approximating the channel geometry to be cylindrical, the potential change (Δ*V*) is given by [10]:


[1] $$\Delta V=\frac{\varepsilon \varepsilon_{0}R\varsigma }{\eta eC\mu }\nu$$

where ε is the dielectric constant of the fluid, ε_0_ is the vacuum permittivity, *R* is the flow resistance of the channel, ζ is the zeta potential of the ionic double layer on the channel surfaces, η is the liquid viscosity, *C* is the ionic concentration, µ is the effective ionic mobility, and ν is the liquid flow rate, which plays an important role in MEGs. The flow potential is a reflection of the joint action of the fluid and the nanochannel, so it can be shown from Equation 1 that various parameters of the nanochannel material, such as flow resistance and surface potential, directly contribute to the flow potential. At the same time, the parameters of the fluid in the nanochannel, such as dielectric constant and ion concentration, are directly related to the magnitude of the flow potential. In the nanochannel, the flow velocity ν of the fluid is related to the rate of charge movement in the nanochannel. This, in turn, is related to the current density in the channel and has a direct contribution to the flow voltage at both ends of the channel. There is a requirement for the nanochannel radius in MEGs, which should be greater than the Debye screening length, about 9 nm for aqueous electrolytes [11–14]:


[2] $$\frac{1}{\kappa }={\left(\frac{\varepsilon_{\text{m}}\varepsilon_{0}k_{\text{B}}T}{2N_{\text{A}}e^{2}C}\right)}^{1/2},$$

where κ is the Debye screening length, ε_m_ is the permittivity of medium, *k*_B_ is the Boltzmann constant, *T* is the temperature, and *N*_A_ is the Avogadro number. In addition, the “two-step” model between liquid and solid proposed by Wang et al. also provides a valuable explanation and deeper understanding of solid–liquid interactions. Compared to the traditional model, Wang’s model suggests that electron transfer between liquid molecules and solid surface atoms is the initial step and is followed by ion transfer due to electronic interaction. The actual charge transfer is much more complicated, involving the contact angle, dielectric function, temperature, and ion concentration [15–17].

In MEGs, compared to bulk materials, nanoarchitectonics yields a higher specific surface area to the active material, which makes the contact area between the nanomaterial and the moisture larger and greatly improves the carrier yield. Therefore, in the abovementioned charge transfer mechanism, nanostructures theoretically yield better performance [18–20]. More researchers have also noticed that nanoarchitectonics can significantly improve the efficiency of energy harvesting in MEGs, and a considerable number of studies have focused on nanomaterials [9,21]. The generation of a flowing current through the injection of water flow into carbon nanotubes was one of the initial studies of MEGs [4–5,10,22–23]. Since then, more works have demonstrated the application of different materials and nanoarchitectonics in MEGs and further improved the performance of the MEGs. The importance and role of nanoarchitectonics have also been gradually verified in these studies.

Nanomaterials can be divided into structural units, such as nanoparticles, nanowires, and nanosheets. In the construction of devices, nanomaterial units are stacked in thin layers or blocks, and gaps are formed between the units, allowing for the formation of nanoscale networks in the stacked regions. When the nanoparticles, nanowires, or nanosheets are stacked in a non-directional selective manner, they form a randomly oriented nanoscale network structure with high surface area, which is convenient for fabrication and beneficial for MEGs. There are various methods to fabricate nanoarchitectonics, including but not limited to electrostatic spinning, lyophilization, photolithography, embossing, deposition, and sol–gel nanofabrication, all of which can provide high specific surface areas [19,24–28].

Nanomaterials can also be divided into inorganic nanomaterials and organic nanomaterials. In inorganic nanomaterials, metal nanomaterials and carbon nanomaterials have superior electrical conductivity and provide better electron transfer properties. Organic nanomaterials are complementary to inorganic nanomaterials in terms of physical properties. Although organic nanomaterials are usually poor in electrical conductivity, they have better properties in terms of biocompatibility and plasticity. Nanoarchitectonics for MEGs can be constructed using organic or inorganic materials alone, or organic–inorganic composite nanomaterials [29–31]. The energy-harvesting component can gather significant amounts of energy for daily use by employing suitable nanomaterials and an appropriate device design that satisfies the carrier transport requirements.

In previous reviews, the physical mechanisms of solid–liquid interactions and MEGs have been systematically discussed [6,32–33], but there is no comprehensive summary of the various types of nanomaterials and nanoarchitectonics used in MEGs. This review provides a comprehensive coverage of the studies in the field of MEGs in terms of nanomaterials, and the connection between the nanoarchitectonics and MEGs will also be introduced. The various parameters that have an influence on the performance of MEGs will be summarized and discussed in detail.

### 2 Inorganic nanomaterials for MEG

#### 2.1 Carbon nanotubes and carbon nanoparticles

Among inorganic nanomaterials, carbon nanoparticles, carbon nanotubes, graphene, graphene oxide, metal oxides, and transition metal chalcogenides (TMDs) have been reported so far regarding applications in MEGs. Among them, carbon materials are favored by researchers due to their good electrical conductivity and mature preparation technologies. In addition, surface modification of materials is an important part of research for MEGs. The surface functional groups of inorganic nanomaterials are easy to control, and the abundant surface charges enable the charge separation of water molecules after the addition of moisture, which contributes to a more efficient generation of electric energy [1–2,34–38].

**Figure 1:**
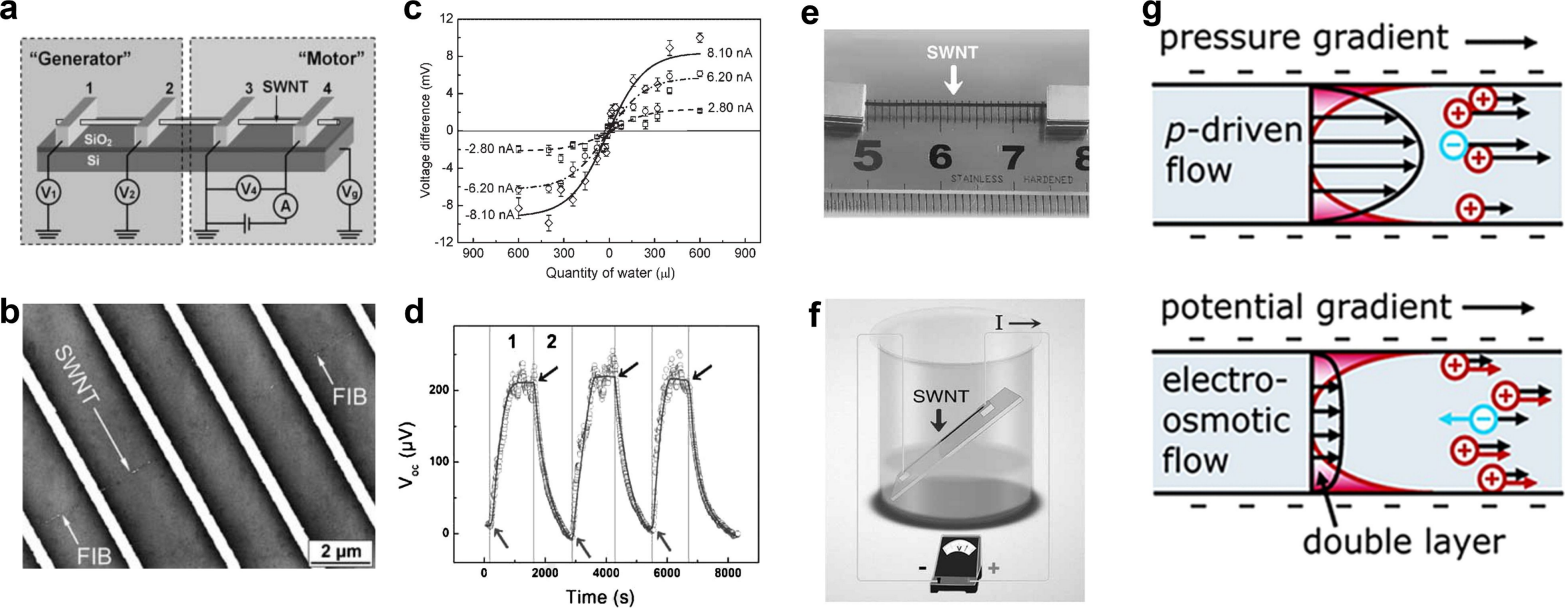
(a) The water flow is driven by an external electric field in the “motor” part, so the water molecules gain kinetic energy, and then an electromotive force is generated in the "Generator" part. (b) A scanning electron microscopy (SEM) image of an individual single-walled carbon nanotube (SWNT) device. (c) Dependence of the induced voltage difference, Δ*V*, on the quantity of water injected into the chamber. Δ*V* increases with the quantity of water inside the chamber and tends to saturate at 500 μL. It is nearly symmetric for either a positive or negative current. Figure 1a–c were reproduced from [7], Y. Zhao et al., “Individual Water-Filled Single-Walled Carbon Nanotubes as Hydroelectric Power Converters”, Adv. Mater., with permission from John Wiley and Sons. Copyright © 2008 WILEY-VCH Verlag GmbH & Co. KGaA, Weinheim. This content is not subject to CC BY 4.0. (d) Single-walled carbon nanotubes convert collected energy into voltage output with repeatability. (e) An image of a suspended SWNT rope. (f) Schematic diagram of an ethanol evaporation energy collection device. Figure 1d–f were reproduced from [44], Liu, Z. et al., “Surface-energy generator of single-walled carbon nanotubes and usage in a self-powered system”, Adv. Mater., with permission from John Wiley and Sons. Copyright © 2010 WILEY-VCH Verlag GmbH & Co. KGaA, Weinheim. This content is not subject to CC BY 4.0. (g) Schematic illustration of electrokinetic effects. Top: A pressure-driven flow carries the net ionic charge within the double layer, generating a streaming current. Bottom: A potential gradient generates both an electro-osmotic fluid flow (black arrows) and an additional electrophoretic ion velocity (colored arrows). Figure 1g was reprinted with permission from [43], Copyright 2007 American Chemical Society. This content is not subject to CC BY 4.0.

In 2003, Ghosh [22] reported that the fluid in single-walled carbon nanotubes can generate an electrical signal up to 10 mV and successfully applied it to a flow sensor. In 2008, Zhao et al. prepared a single-walled carbon nanotube generator [7], which imparted momentum to the water in the carbon nanotubes by applying a voltage to both ends of the carbon nanotubes, such that the other part of the single-walled carbon nanotube generated a millivolt-level voltage and nanoampere-level current (Figure 1a). These early confirmatory studies demonstrate that the liquid flow potential can be harnessed to generate electrical energy (Figure 1c,d). They also play a basic role in further exploiting the flow potential in nanochannels [34,39–44].

**Figure 2:**
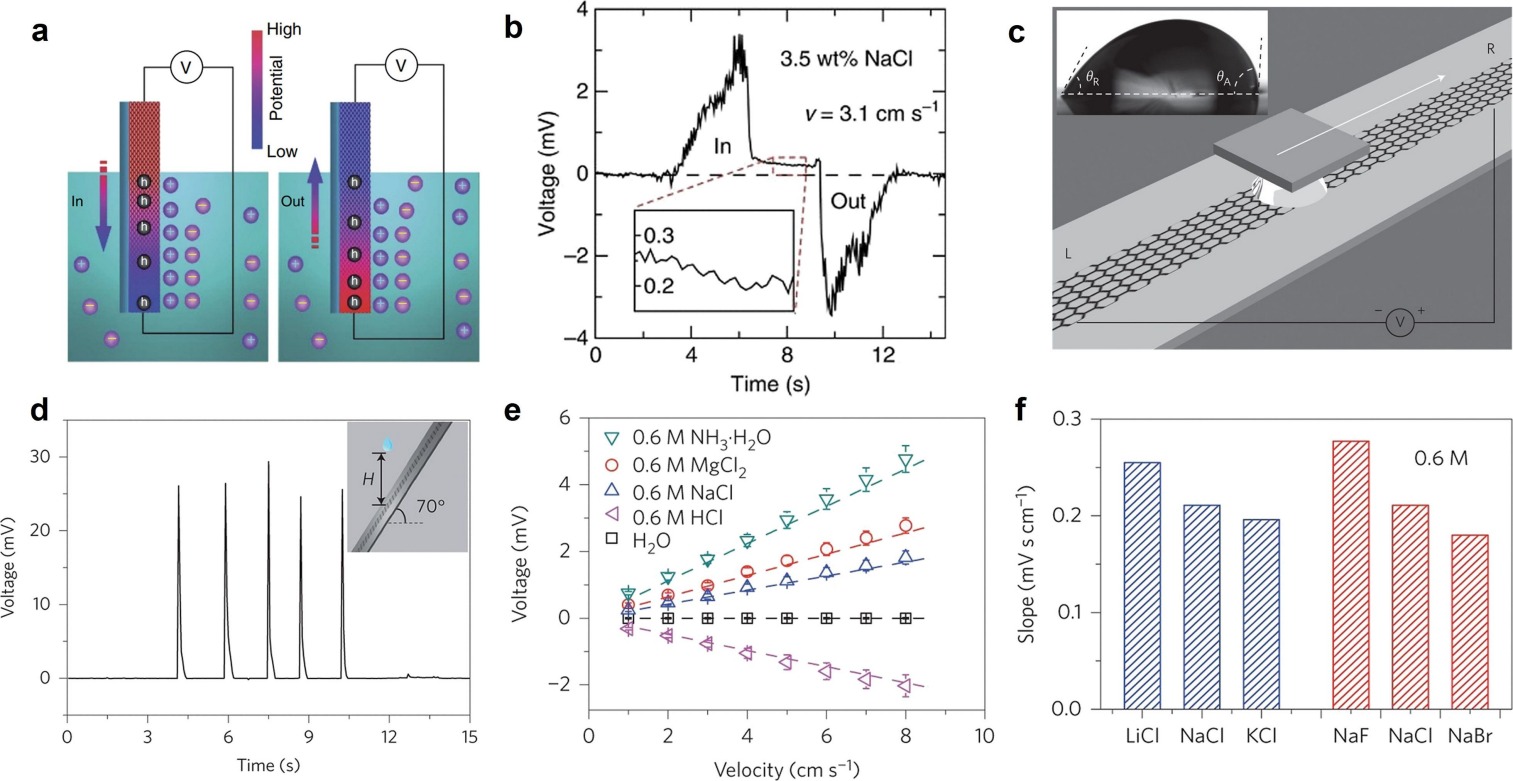
(a) Schematic diagrams of the single-layer graphene device and wave energy harvesting. (b) The voltage signal produced by inserting and withdrawing the sample at *v* = 3.1 cm/s in 0.6 M NaCl (3.5 wt %) solution. Figure 2a and Figure 2b are from [45] and were reprinted by permission from Springer Nature from the journal Nature Communications (“Waving potential in graphene“ by J. Yin; Z. Zhang; X. Li; J. Yu; J. Zhou; Y. Chen; W. Guo), Copyright 2014 Springer Nature. This content is not subject to CC BY 4.0. (c) A liquid droplet is sandwiched between graphene and a SiO_2_/Si wafer and is drawn at specific velocities by the wafer. (d) A pulse voltage is generated by continuously falling droplets. (e) Voltage induced by three droplets of different solutions. (f) Fitted slope *A* = *V*/*v* (*V* of voltage, *v* of velocity) for three droplets of different chloride and sodium salts. Figure 2c–f are from [8] and were reprinted by permission from Springer Nature from the journal Nature Nanotechnology (“Generating electricity by moving a droplet of ionic liquid along graphene“ by J. Yin; X. Li; J. Yu; Z. Zhang; J. Zhou; W. Guo), Copyright 2014 Springer Nature. This content is not subject to CC BY 4.0.

In 2014, Jun Yin et al. harvested wave energy using a single-layer graphene sheet [8,45]. The device produced a detectable electrical signal between the upper and lower electrodes when the graphene sheet was not fully immersed in the solution and was moved vertically. The electrical output signal was about 60 mV and 4 μA (Figure 2a,b). Yin’s research group also studied the voltage response of graphene layers to moving droplets [34], and the variables in the experiment included the number of graphene layers, droplet size, and ion species. Dropping a droplet of 80 μL 0.6 M CuCl_2_ solution from 15 cm above the contact point onto a 70° tilted graphene surface under gravity may create a pulse voltage of 30 mV and a short-circuit current of 1.7 μA, as shown in Figure 2c–f.

**Figure 3:**
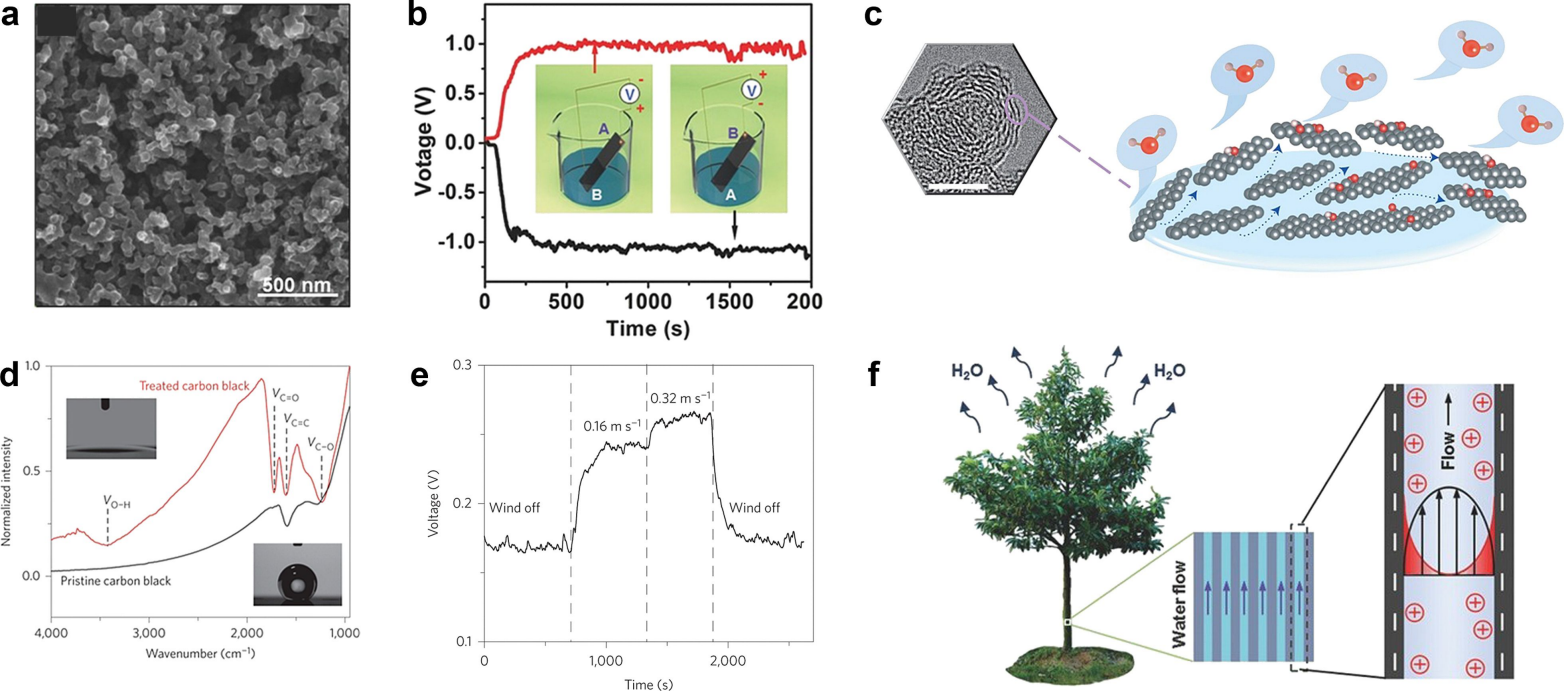
(a) SEM image of the porous carbon film. (b) The porous carbon film power generation device and its performance are depicted schematically. Figure 3a, 3b, and 3f were reproduced from [9], Ding, Tianpeng et al., “All-Printed Porous Carbon Film for Electricity Generation from Evaporation-Driven Water Flow”, Adv. Funct. Mater., with permission from John Wiley and Sons. Copyright © 2017 WILEY-VCH Verlag GmbH & Co. KGaA, Weinheim. This content is not subject to CC BY 4.0. (c) HR-TEM image of CB and a schematic depiction of water evaporation and induced water flow in CB. (d) After annealing and air plasma cleaning, changes in hydrophilicity and functional groups of the porous carbon film. (e) Voltage between the two electrodes at different wind velocities. Figure 3c, 3d, and 3e are from [46] and were reprinted by permission from Springer Nature from the journal Nature Nanotechnology (“Water-evaporation-induced electricity with nanostructured carbon materials“ by G. Xue; Y. Xu; T. Ding; J. Li; J. Yin; W. Fei; Y. Cao; J. Yu; L. Yuan; L. Gong; J. Chen; S. Deng; J. Zhou; W. Guo), Copyright 2017 Springer Nature. This content is not subject to CC BY 4.0. (f) Schematic diagram of the liquid flow-induced streaming potential in a natural microchannel.

Carbon nanoparticles form nanoscale networks by stacking deposition (Figure 3a). Zhou Jun's group used this kind of carbon nanoscale network to absorb the evaporation energy of water vapor and have a stable electrical output of 1 V, 100 nA in 2017 [9,37,46]. The carbon nanoparticles are easy to obtain, and a large number of carbon nanoparticles can be collected from the residual soot after flame combustion of an organic liquid. After annealing and air plasma cleaning, the surface of these nanoparticles becomes hydrophilic, resulting in water molecules moving from the bottom to the top through capillary action in a natural water evaporation environment. The induced voltage was detected from the top and lower electrodes during movement. Annealing treatment and plasma cleaning of the carbon particle layer have significant effects on the output voltage. After the treatment, the material changes from hydrophobic to hydrophilic, the contact angle decreases from 143.25° to 10.15° (Figure 3d), and the contact area between carbon nanomaterials and water molecules is greatly increased. This leads to a stronger interaction between water molecules and the materials. Also, the treated carbon nanoparticles possess a large number of functional groups with O–H, C–O and C=O, C–OH, C–O–C, and O=C–OH bonds. The voltage increased from 45 μV to 1 V after increasing the liquid–solid contact area and the fraction of functional groups, proving the important role of these parameters in MEGs.

These carbon nanomaterial MEG devices are an important part of the MEG field. First, carbon nanoparticles are easy to obtain and can provide a rich specific surface area by simple stacking. Second, many important parameters of MEGs, such as fluid velocity and liquid ion concentration, have been mentioned first in studies related to carbon nanotubes and carbon nanoparticles, which have contributed significantly to the development of the principle exploration of MEG. Future research work should be inspired by these proof-of-principle studies to develop innovative advances.

#### 2.2 Metal compounds

**Figure 4:**
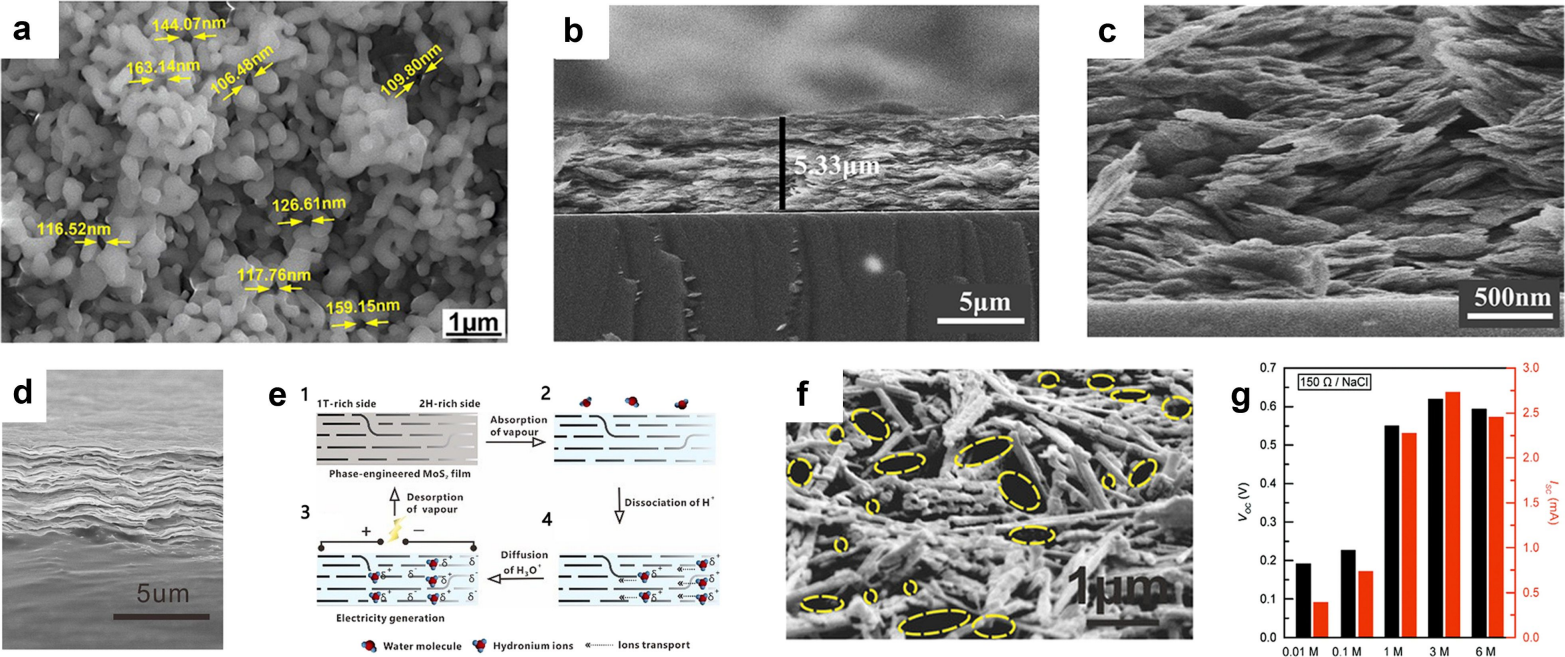
(a) Surface morphology of an Al_2_O_3_ layer. Figure 4a was reprinted with permission from [49], Copyright 2021 American Chemical Society. This content is not subject to CC BY 4.0. (b, c) Cross-sectional SEM images of a Ni–Al LDH film. Figure 4b and 4c were adapted from [52]. This article was published in Nano Energy, vol. 70, by J. Tian; Y. Zang; J. Sun; J. Qu; F. Gao; G. Liang, “Surface charge density-dependent performance of Ni–Al layered double hydroxide-based flexible self-powered generators driven by natural water evaporation“, article no. 104502, Copyright Elsevier (2020). This content is not subject to CC BY 4.0. (d) A cross-sectional SEM image of the phase-engineered MoS_2_ film with a film thickness of 4.6 µm. (e) The adsorption and desorption process of ions under the phase gradient of MoS_2_. Figure 4d and 4e were reprinted from [50]. This article was published in Nano Energy, vol. 81, by D. He; Y. Yang; Y. Zhou; J. Wan; H. Wang; X. Fan; Q. Li; H. Huang, “Electricity generation from phase-engineered flexible MoS2 nanosheets under moisture“, article no. 105630, Copyright Elsevier (2021). This content is not subject to CC BY 4.0. (f) SEM image of high porosity TiO_2_ nanowire network. Figure 4f was reproduced from [54], Shen, D. et al., “Self-Powered Wearable Electronics Based on Moisture Enabled Electricity Generation”, Adv. Mater., with permission from John Wiley and Sons. Copyright © 2018 WILEY-VCH Verlag GmbH & Co. KGaA, Weinheim. This content is not subject to CC BY 4.0. (g) Measured *V*_OC_ and *I*_SC_ values from the MXenes TEPG (transpiration-driven electrokinetic power generators) with NaCl solutions of various concentrations. Figure 4g was reproduced from [55] (“Towards Watt-scale hydroelectric energy harvesting by Ti_3_C_2_T*_x_*-based transpiration-driven electrokinetic power generators“, © 2022 J. Bae et al., published by Royal Society of Chemistry, distributed under the terms of the Creative Commons Attribution-NonCommercial 3.0 Unported License, https://creativecommons.org/licenses/by-nc/3.0/). This content is not subject to CC BY 4.0.

Metal oxide and transition metal chalcogenide materials perform similarly in MEGs. These materials can be easily grown on substrates to form nanoscale networks and perform well in MEGs due to their unique electron transport properties [2,47–48]. This includes Al_2_O_3_ [49], MoS_2_ [50–51], Ni–Al layered double hydroxide (LDH) [52], MoS_2_/SiO_2_ composites [53], TiO_2_ [54], and Ti_3_C_2_T*_x_* MXene nanosheets [55]. A hydroelectric conversion device prepared by suction filtration of 200 nm Al_2_O_3_ nanoparticles provided an instantaneous electrical output of 4 V and 18 μA (Figure 4a). The Ni–Al layered double hydroxide material has a high specific surface area and provided a constant electrical output with a maximum *V*_oc_ of 0.6 V and an *I*_sc_ of 0.3 μA (Figure 4b,c). TiO_2_ nanowires provided an open-circuit voltage of 0.5 V and microampere-level short-circuit currents through a rich 3D nanoscale network. In a Ti_3_C_2_T*_x_* MXene nanosheet generator, the wicking rates of the samples after sonication were increased, and the device provided an instantaneous electrical output with a maximum open-circuit voltage of 0.3 V and a short-circuit current of 120 μA. After adding a conducting polymer to improve the number of charged ions and the ion selectivity of the diffusion channel, a specific electrolyte fluid was selected to interact with the nanochannel. The resulting voltage and current values were increased by 2.5 times and 19 times, respectively, to 0.69 V and 7.55 mA using NaCl solution. Such high current outputs are rarely reported in transient output devices. Therefore, this work deserves continuous attention. A phase-engineered flexible MoS_2_ nanosheet generator is worth mentioning. Through annealing at 150 °C, the 1T phase of MoS_2_ is changed to the 2H phase. The 2H phase can dissociate more water molecules into hydrogen ions than the 1T phase (Figure 4d,e). Thus, the different phases yield a difference in hydrogen ion concentration and this difference forms the induced current in the external circuit. The MoS_2_ film can provide a continuous electrical output of 19 mV and 6.24 μA. The introduction of ion concentration difference is a valuable improvement. This artificially created internal ion concentration difference can significantly improve the electrical output performance. We will also see more applications and manifestations of this improvement in the following work.

Among the metal compound nanomaterials, a Ti_3_C_2_T*_x_* MEG device had a maximum transient current of 7.55 mA in salt solution, which is the highest instantaneous current measured in nanomaterial MEG devices. Moreover, the preparation method of metal compound nanomaterials is mature, and different morphologies can be obtained, such as nanoparticles of Al_2_O_3_, nanowires of TiO_2_, Ni–Al layered structures, and nanosheets of MXene. Different nanostructures also have an influence on the performance of MEGs, which is worth further investigation. Metal compound nanomaterials have been successfully used in many fields, such as optoelectronic, thermoelectric, and piezoelectric devices [56–60]. With the knowledge about preparation methods and fundamental physical and chemical properties, it is expected that more metal compounds will be used in the field of MEGs and help to create devices with higher performance.

#### 2.3 Graphene

**Figure 5:**
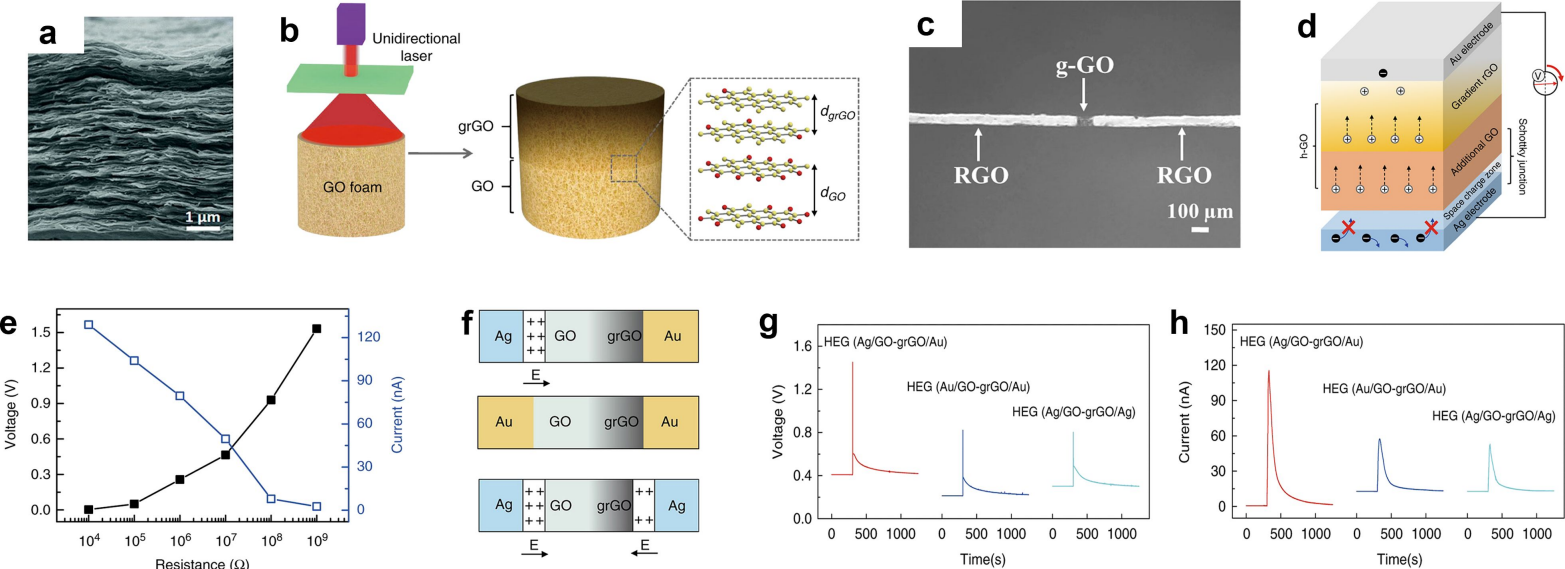
(a) Cross-sectional SEM image of GO’s nanoscale network structure. Figure 5a was republished with permission of The Royal Society of Chemistry from [63] (“Electric power generation via asymmetric moisturizing of graphene oxide for flexible, printable and portable electronics” by Y. Liang et al., Energy Environ. Sci., vol. 11, issue 7, © 2018); permission conveyed through Copyright Clearance Center, Inc. This content is not subject to CC BY 4.0. (b) Schematic diagram of the structure of vertically oriented asymmetric graphene. (c) Image of laser-processed asymmetric graphene fibers. Figure 5c was reprinted from [62]. This article was published in Nano Energy, vol. 32, by Y. Liang; F. Zhao; Z. Cheng; Q. Zhou; H. Shao; L. Jiang; L. Qu, “Self-powered wearable graphene fiber for information expression“, pages 329–335, Copyright Elsevier (2017). This content is not subject to CC BY 4.0. d) The device is designed through the interface to increase the output power. (e) Voltage and current output of the device. (f) Device configurations with different electrode designs. (g, h) Voltage and current output of three generators with different device configuration. Figure 5b,d–h were reproduced from [64] (© 2018 Y. Huang et al., published by Springer Nature, distributed under the terms of the Creative Commons Attribution 4.0 International License, https://creativecommons.org/licenses/by/4.0).

Liang-Ti Qu's group introduced a difference in ion concentration to fabricate a series of MEGs with hygroscopic graphene oxide (GO) [61–65]. As shown in Figure 5, when the graphene oxide bulk material with a certain thickness is irradiated by a laser, the laser intensity inside the graphene is gradually attenuated, so that the oxygen-containing functional groups and carbon atom concentration of graphene oxide on the outer side are higher and more hydrophilic than the part that did not receive laser irradiation (Figure 5b). After exposure to moisture, a significant difference in ion concentration occurs due to the difference in humidity and the ability to dissociate hydronium ions, thereby driving ions to move in a direction and providing an output voltage. Graphene is a layered two-dimensional nanomaterial and can yield a large specific surface area by stacking. The method of creating gradient distributions with lasers is quite flexible and practical and works well in both structures of wire-shaped and bulk materials (Figure 5b,c). More importantly, the design of the gradient distribution structure mentioned is an important part of the asymmetric ion diffusion [57,66–67].

### 3 Organic nanomaterials for MEG

#### 3.1 Polyethylene derivatives

**Figure 6:**
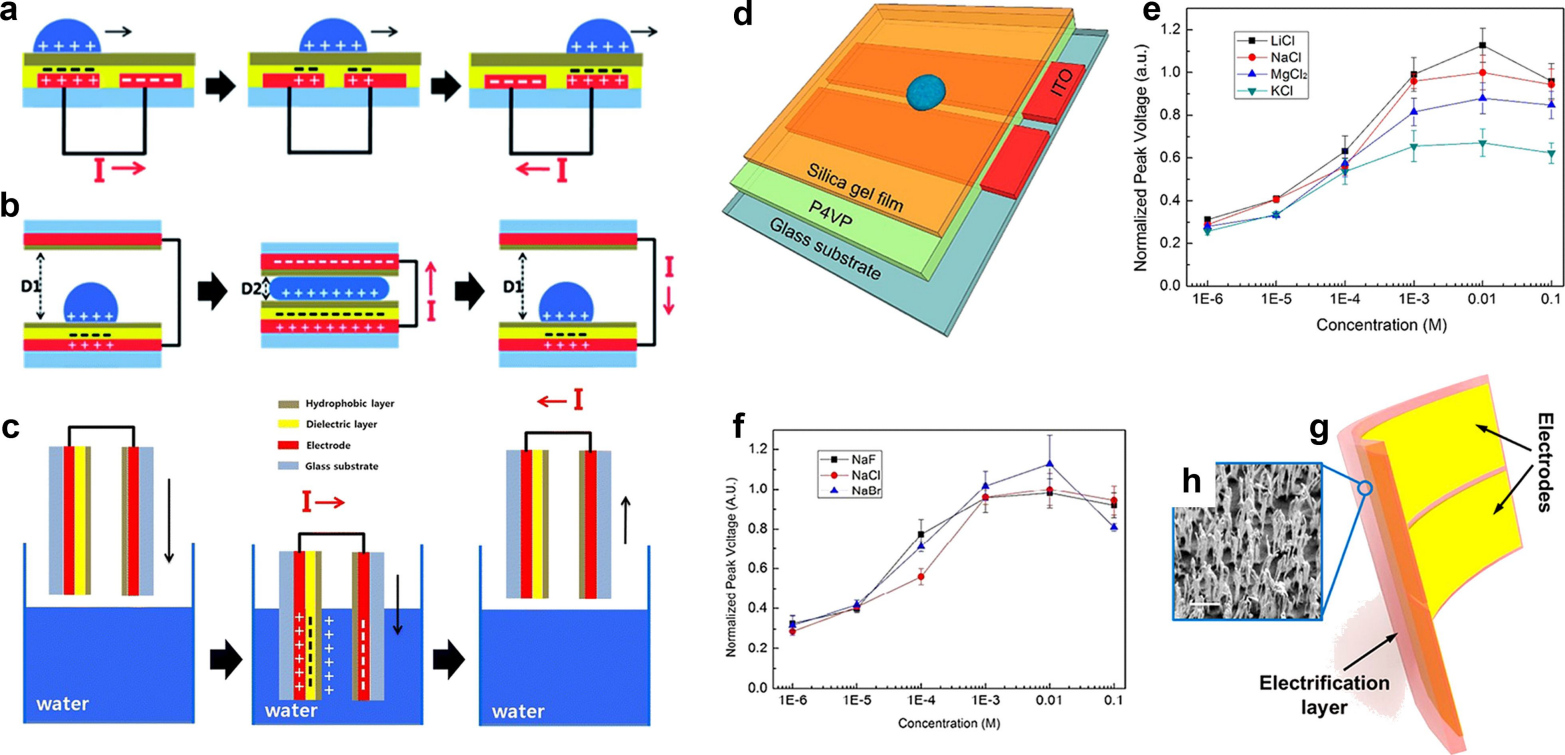
Design and mechanism for (a) flat type, (b) pushing/releasing type, and (c) dipping type (c) of water motion active transducers. Figure 6a–c were republished with permission of The Royal Society of Chemistry from [75] (“An effective energy harvesting method from a natural water motion active transducer” by Kwon, Soon-Hyung et al., Energy Environ. Sci., vol. 7, issue 10, © 2014); permission conveyed through Copyright Clearance Center, Inc. This content is not subject to CC BY 4.0. (d) Schematic image of the F4VP device. (e) Using chloride ions as the fixed anions, various alkali metal ions were used to study the effect of the atomic number of the cation on the output power. (f) Using sodium ions as fixed anions, various alkali halide anions were used to study the effect of the atomic number of the anion on the output power. Figure 6d–f were reprinted with permission from [78], Copyright 2015 American Chemical Society. This content is not subject to CC BY 4.0. (g) Structural design of a FEP device. (h) SEM image of the polymer nanowires on the polymer layer. The scale bar is 1 μm. Figure 6g and 6h were reprinted with permission from [21], Copyright 2014 American Chemical Society. This content is not subject to CC BY 4.0.

In recent years, preparation and application of organic materials have been developed rapidly in many fields, including optoelectronic devices, actuators, sensors, and water purification films [18,27,29,31,68–74]. The electrical output of MEG devices with different nanomaterials is listed below in Table 1. A wide range of materials is available in MEGs, such as polymers, proteins, and natural fibers, poly(4-vinylphenol) (PVP) [75], polyvinyl alcohol (PVA) [76–77], fluorinated ethylene propylene (FEP) [21], and polyvinylidene difluoride (PVDF). These materials show good performance in the application of hydropower conversion. In addition, there are also some novel innovations in energy-harvesting structures, such as the construction of lateral gradients of nanomaterials, the construction of multilayer structures, and the direct use of ion distribution gradients in liquids. With a PVP thin film of 290 nm thickness as the dielectric layer, a device designed for collecting natural wave energy obtains an instantaneous electrical output of 6 V and 70 μA [75]. Three structural designs for collecting energy, namely flat type, pushing/releasing type, and dipping type are presented in Figure 6a–c. The P4VP device is designed to collect the energy from the movement of water droplets and provides a 3 V, 2 μA instantaneous electrical output. It is designed as a hydrophobic layer plus a polymeric dielectric layer with an electrode layer for electron flow at the bottom [78]. As shown in Figure 6e and f, the effects of different ions on the output performance of the device was investigated. In this material, chloride ions are immobilized as an anion, and the output performance of the device decreases as the atomic number of the cation increases, this conclusion is the same as the experiment in the graphene work [8]. However, when the halide ion is used as the transition ion and the sodium ion is used as the fixed cation, the output performance of the device does not change much. These experimental results show that in nanochannels, only specific ions can interact with the channel material interface to convert their own flow potential. However, after the solid–liquid contact, the research on the principle of the selective interaction of ions at the solid interface is still not comprehensive and systematic. And more experimental work is needed to complement and prove it.

In 2014, Zhu et al. fabricated a nanowire fluorinated ethylene propylene (FEP) film and made it have a nanoscale network structure with rough surface as shown in Figure 6g,h [21]. On a fluctuating water surface, the upper and lower electrodes on the back of the polymer will generate a potential difference, thereby generating current and voltage. Multiple device groups are connected in parallel to produce a transient output with a voltage of 160 V and a current of 10 μA. When it is directly used to power LED lights, the power generation device group can make 10 LEDs flash for a moment. Increasing the fluctuation frequency can make the LEDs flash continuously. The macroscopic potential difference generated in a nanoscale network structure by a flowing current is also called a liquid–solid triboelectric generator. The effect of surface modification of the nanostructure is evident, with devices with nanowires yielding a 50% increased induced charge compared to devices without modification. The high surface area from the nanowire arrays perpendicular to the surface plays an important role in increasing the output power.

MEG devices based on polyethylene derivatives have been prepared using a variety of methods to obtain nanoscale networks with high porosity, including spin-coating, lyophilization, and electrostatic spinning. These devices using polyethylene derivatives generate high output voltages and can be fabricated at a large scale, which gives them a great application potential. For example, FEP devices generated a high output voltage of up to 160 V with multiple device connections in parallel and series, and polyethylene derivative composites can be fabricated as large-area textiles [77]. Therefore, it is expected that there will be more studies on the application of polyethylene derivative MEGs.

#### 3.2 Nanocellulose

**Figure 7:**
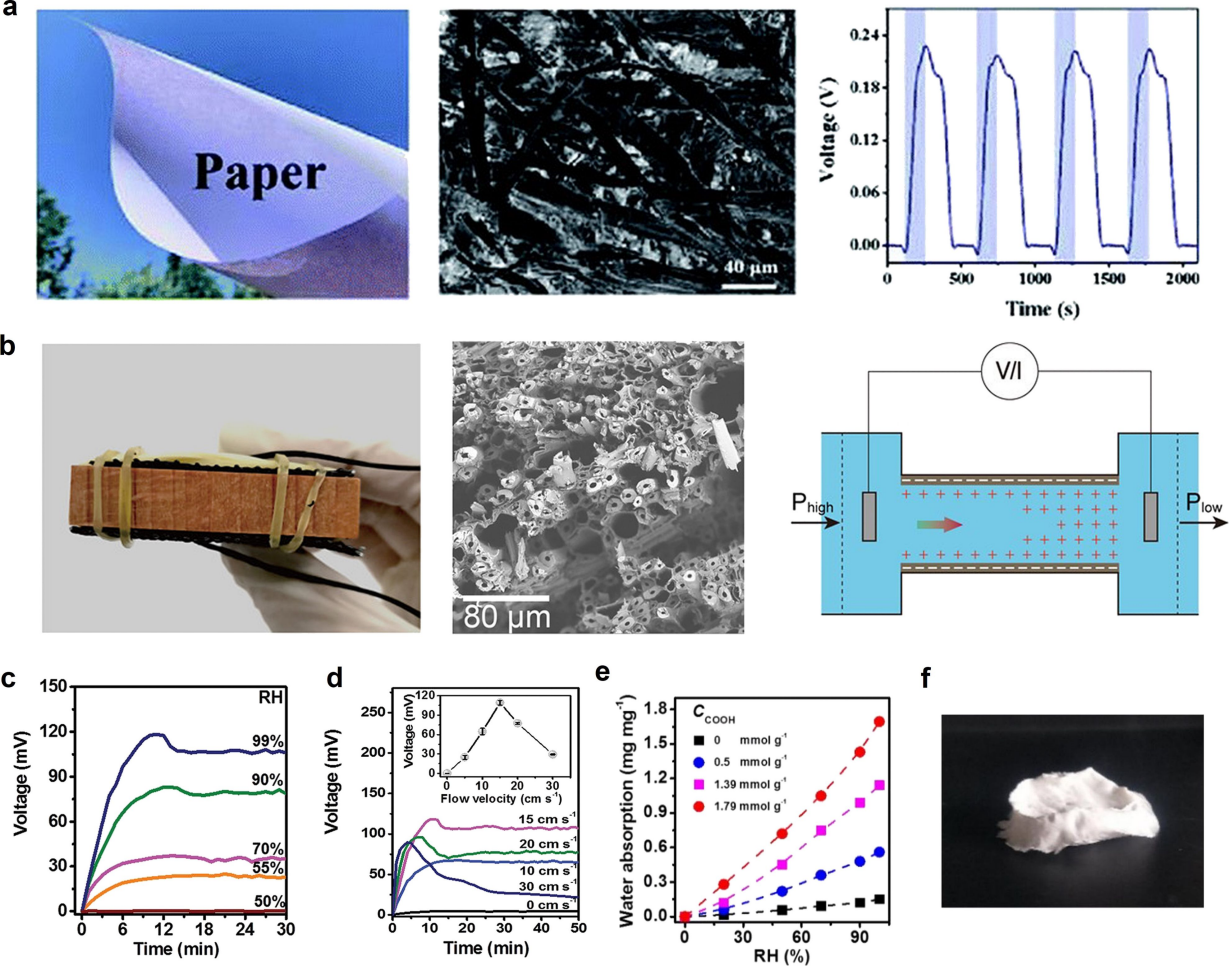
(a) From left to right: a photo of a sheet of paper, a SEM image of a paper sheet, and the voltage generated by moisture on the paper. Figure 7a was republished with permission of The Royal Society of Chemistry from [79] (“Electric power generation using paper materials” by Gao, Xue et al., Journal of Materials Chemistry A., vol. 7, issue 36, © 2019); permission conveyed through Copyright Clearance Center, Inc. This content is not subject to CC BY 4.0. (b) A picture of a wood nanogenerator, a SEM image of beech wood, and a schematic illustration of the device are shown from left to right. Figure 7b was reprinted with permission from [81], Copyright 2020 American Chemical Society. This content is not subject to CC BY 4.0. (c) *V*_OC_ variation upon exposure to air flow at different RH values, flow velocity: 15 cm·s^−1^. (d) *V*_OC_ variation upon exposure to air flow with different velocities, RH: 99%. (e, f) Water absorption of samples with different hydroxy group content and structural collapse of samples with high hydroxy group content. Figure 7c–f were reproduced from [80], Li, Mingjie et al., “Biological Nanofibrous Generator for Electricity Harvest from Moist Air Flow”, Adv. Funct. Mater., with permission from John Wiley and Sons. Copyright © 2019 WILEY-VCH Verlag GmbH & Co. KGaA, Weinheim. This content is not subject to CC BY 4.0.

Among organic materials, nanocellulose is commonly used because cellulose is easy to obtain. The main component of plant cell walls in nature is cellulose, and more than 50% of the carbon content of plants in nature is cellulose. Cotton contains almost 100% cellulose, therefore, cellulose is abundant in nature and can be used to fabricate MEG devices at low cost. In addition, due to the high porosity and large specific surface area of cellulose-based materials, various surface functional groups can be introduced. Thus, cellulose is an excellent hydroelectric conversion material. In addition to the abovementioned work, existing work includes materials such as paper [79], cellulose nanofibrils, which were exfoliated mechanically from naturally biomass [80], natural wood [81]. In other works with cellulose doping [55,82–83], voltages of, respectively, 250, 100, and 300 mV were obtained. Li et al. studied in detail the effects of various parameters such as humidity, airflow rate, airflow direction, and number of oxygen-containing groups on the output power of nanostructures [80]. Figure 7c shows that the higher the humidity, the greater the output voltage. When the relative humidity of the device increases, the interaction area between the material and the water molecules will increase, or in the case of a constant flow rate, more nanochannels participate in the conversion of streaming potential energy, increasing the electrical output. But is there an upper limit to the effect of humidity on the output power for different materials? Also, how the largest possible proportion of nanochannels participates in the interaction with water molecules in a completely liquid environment is a problem for further exploration. The airflow rate is an important parameter for the output power (Figure 7d). From Equation 1, we can see that the faster the water molecules move on the surface of the material (*ν*), the higher the output voltage (Δ*V*) [10]. This conclusion was also drawn in reports regarding carbon nanomaterials [9]. The fluid in the real device will also evaporate while being absorbed. Hence, theoretically there will be a dynamic balance between absorption and evaporation. Whether there is an upper limit for the dynamic balance is not clear for the time being, but the qualitative conclusion conforms to basic logic and is still used as a guiding principle for improving the output power. In terms of airflow direction, the output voltage is the highest under a moisture airflow parallel to the nanostructure direction. If the structure direction is perpendicular to the airflow, the voltage is the lowest. The output voltage of a non-directional nanoscale network structure under airflow is at a medium level. In comparison with directional nanochannel structures, non-directional nanostructures have a stronger resistance to the airflow direction and can maintain a good output voltage level regardless of the airflow direction. But when a directional structure is parallel to the airflow, the output voltage can be increased under suitable conditions. It is reasonable to assume that a higher voltage output is achieved by increasing the flow velocity of water molecules in the nanoscale network [9,22]. The last thing to discuss is the effect of the fraction of oxygen-containing groups on the output power (Figure 7e). The output power of samples was significantly increased by the addition of oxygen-containing groups [50,64,84]. More functional groups represent lead to a higher ion content. Thus, the flowing liquid in the microchannel will carry more diffusion layer ions, increasing the output voltage. Li et al. [68] studied the effect of the fraction of hydroxy groups on the output power. The was a generally positive correlation. However, an excess of hydroxy group content as shown in Figure 7f may affect the stability of the nanostructure and lead to the collapse of the structure.

Cellulose materials have great application potential in MEGs due to their rich natural composition. Furthermore, functional groups can easily be added to cellulose-based MEGs to improve the output power.

#### 3.3 Other polymer nanomaterials

**Figure 8:**
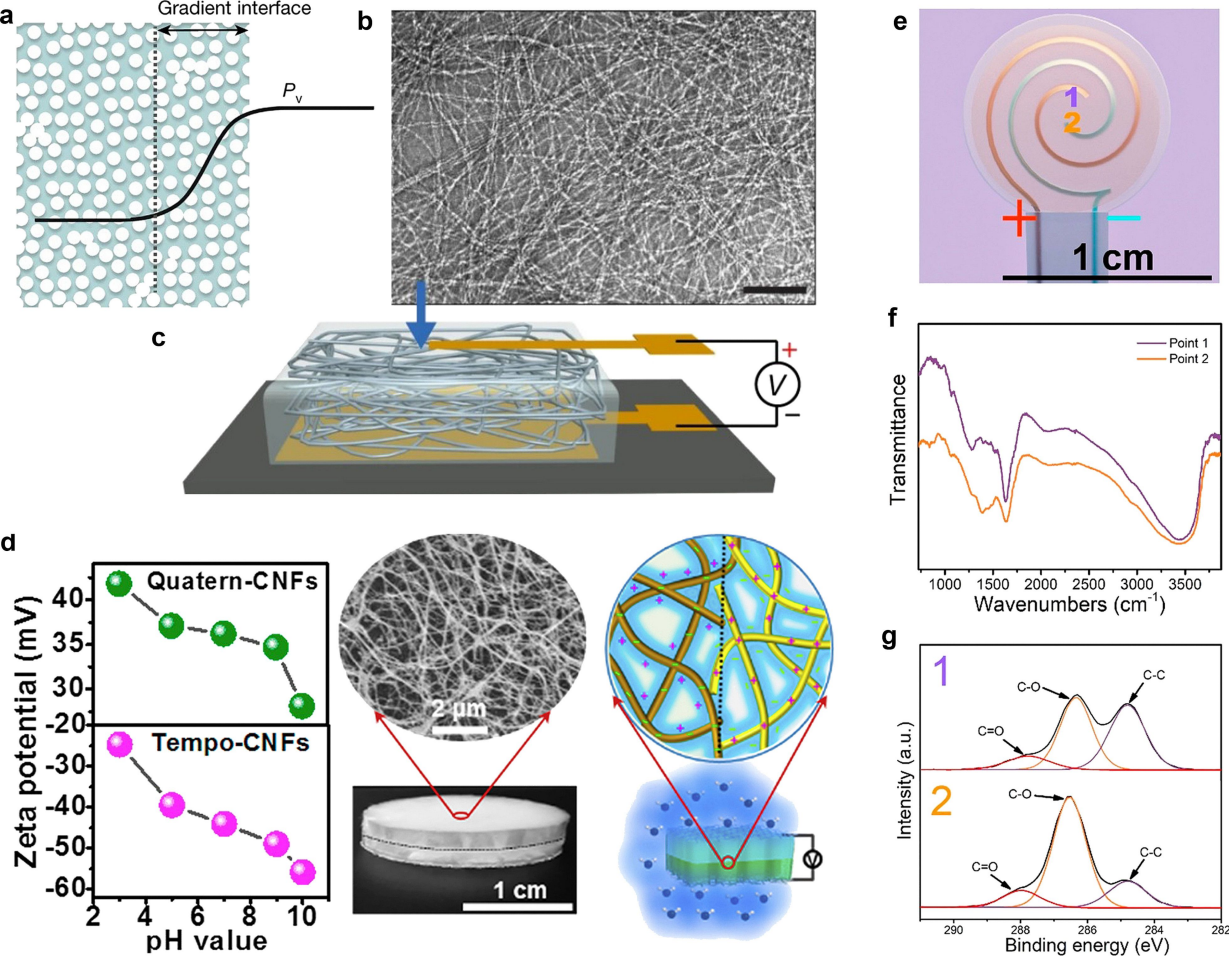
(a) Vapor pressure gradient near the air interface. (b) TEM images of the purified nanowire network. Scale bars: 100 nm. (c) Diagram of the protein device structure. Figure 8a–c are from [86] and were adapted by permission from Springer Nature from the journal Nature (“Power generation from ambient humidity using protein nanowires“ by X. Liu; H. Gao; J. E. Ward; X. Liu; B. Yin; T. Fu; J. Chen; D. R. Lovley; J. Yao), Copyright 2020 Springer Nature. This content is not subject to CC BY 4.0. (d) From left to right are the zeta potential values of two different CNFs, the SEM image of the nanostructure, and the schematic diagram of the heterostructure. Figure 8d was adapted from [85]. This article was published in Nano Energy, vol. 71, by W. Yang; X. Li; X. Han; W. Zhang; Z. Wang; X. Ma; M. Li; C. Li, “Asymmetric ionic aerogel of biologic nanofibrils for harvesting electricity from moisture“, article no. 104610, Copyright Elsevier (2020). This content is not subject to CC BY 4.0. (e) Schematic diagram of a fabricated power generator with a g-PDA film and two spiral electrodes for (1) the anode and (2) the cathode. (f) Infrared spectra of g-PDA near (1) the anode and (2) the cathode. (g) High-resolution C 1s spectra of the g-PDA film for (1) the anode and (2) the cathode. Figure 8e–g were reprinted with permission from [84], Copyright 2019 American Chemical Society. This content is not subject to CC BY 4.0.

When an ion concentration gradient is generated in the nanostructure, this gradient distribution makes ions move from regions of high concentration to regions of low concentration. This enhances the transfer of electric charges and improves the electrical energy output [61]. Under natural conditions, a film material with a certain thickness will induce a difference in water concentration due to its poor adsorption capacity in different directions, which naturally leads to a difference in the concentration of ions contained in the film material. These film materials include biological nanofibers (NFs) [85], porous polydopamine (g-PDA) [84], protein nanowires [86], and gelatin molecules [87], which yielded output voltages of 0.115, 0.52, 0.5, and 0.71 V, respectively. As shown in Figure 8a, the existence of this natural concentration difference has been experimentally proved in a work regarding protein nanowires. In organic materials, in addition to the difference in natural adsorption capacity to construct gradient structures, gradient structures can also be artificially fabricated. For example, in 2020, Yang et al. measured a sustained voltage from a heterogenized device in the vertical direction using two materials, namely Quatern-CNFs (top) and TEMPO-CNFs (bottom) (Figure 8d). Using g-PDA, by applying a voltage to two spiral electrodes, the material between the electrodes generated a gradient of hydroxy groups through polarization. The cathode was reduced to generate more hydroxy groups; the anode was oxidized to generate more o-quinone structures. This asymmetry was demonstrated by the stronger hydrogen bonding near the cathode, measured by infrared spectroscopy (Figure 8f,g). Under moist conditions, a voltage signal could be measured in the gradient structure device, while the PDA film without polarization treatment did not produce an obvious voltage or current signal. This lateral gradient configuration is as efficient as most vertical gradient configurations and can yield a considerable electrical output. This also opens up ideas for the design of MEGs adopted to different usage conditions or environments. The device structure can be flexibly selected to achieve functional purposes, and the gradient structure can be constructed relative to any direction in space to meet the needs.

**Figure 9:**
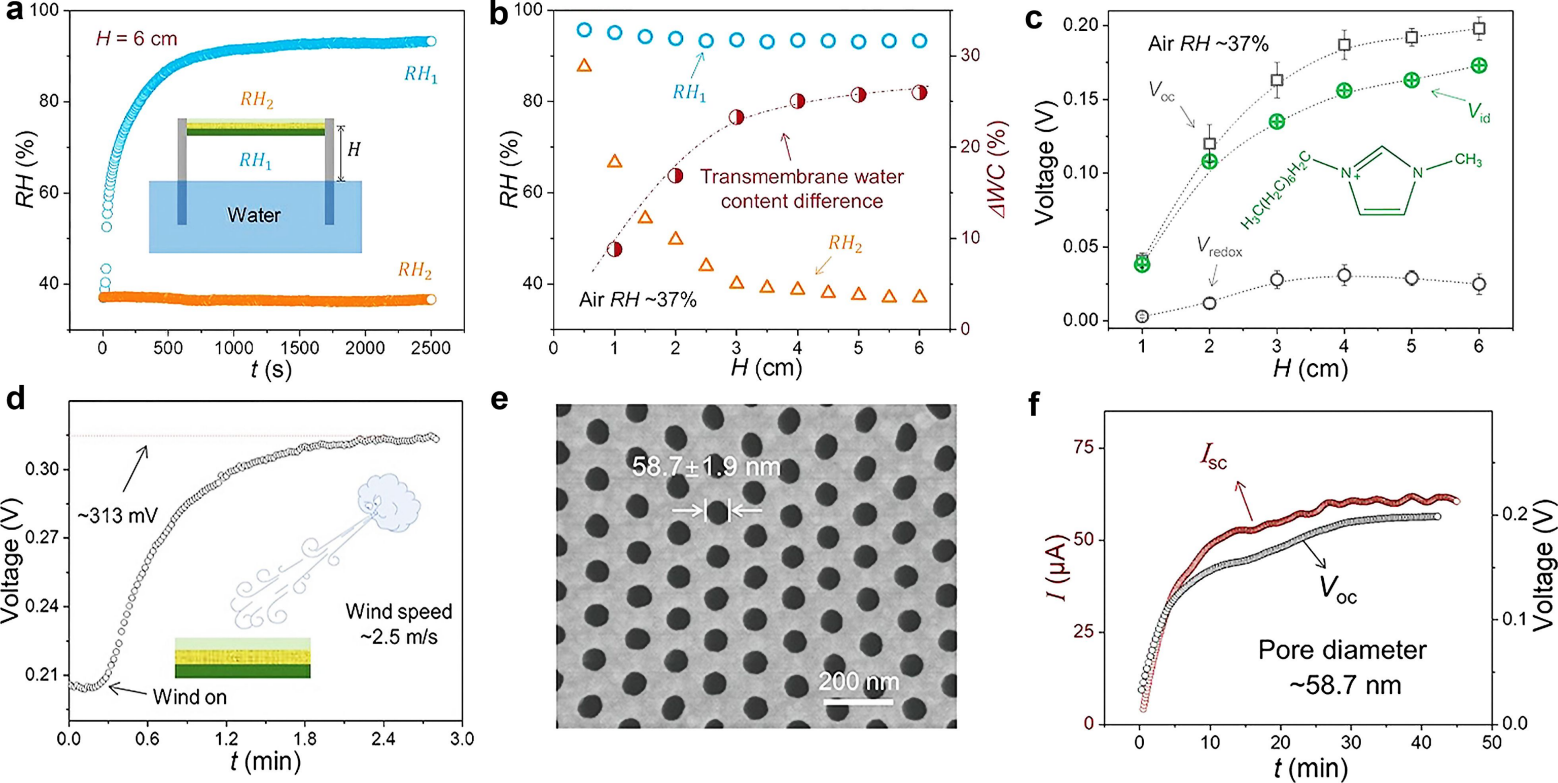
(a) RH at both sides of the membrane. *H* = 6 cm. (b) RH_1_ and RH_2_ as functions of *H*. ΔWC is the transmembrane water content difference. (c) *V*_OC_ and *V*_id_ as functions of *H*. (d) Air flow (2.5 m·s^−1^)-enhanced power generation. (e) SEM image of the prepared ion-selective membrane (pore size diameter ≈ 58.7 nm). (f) *V*_oc_ and *I*_sc_ as functions of the time. Figure 9a–f were reproduced from [88], Zheng, S. et al., “Continuous Energy Harvesting from Ubiquitous Humidity Gradients using Liquid-Infused Nanofluidics”, Adv. Mater., with permission from John Wiley and Sons. Copyright © 2021 WILEY-VCH GmbH. This content is not subject to CC BY 4.0.

Moisture-electric conversion devices in nanofluids have also been recently reported. An ionic liquid film of Omim^+^ Cl^−^ was selected as the ion-exchange layer [88]. In a moisture-gradient environment (Figure 9) on both sides of the ionic liquid layer a humidity difference was constructed. Ions are driven by the ion concentration difference and then pass through the membrane by selective ion diffusion. The ion-selective membrane is designed to hinder specific ions from passing through the membrane such that a potential difference between the two sides of the membrane can be created The thickness and the size of the nanochannels of this membrane will influence the effect of asymmetric ion diffusion. This asymmetric ion diffusion can produce a maximum electrical output of 0.3 V and 0.1 μA. The selections of ionic liquids, the selection of ion-permeable membranes, and the effects of surface functional groups are discussed in detail in the paper and will not be repeated here. By adding an ion-selective membrane, ion concentration diffusion can take place in the fluid, not just in the bulk material. This innovation broadens the design ideas for moist-electric conversion devices.

**Table 1:** Electrical output of MEG devices with different nanomaterials.

	active materials	*V*_OC_	*I*_SC_	Wattage	Mode	ref.

inorganic nanomaterials	graphene	0.06 V	4 μA	0.24 μW	instantaneous	[16]
	carbon nanotubes	0.008 V	8 nA	0.064 nW	instantaneous	[7]
	carbon nanoparticles	0.9 V	0.6 μA	0.588 μW	instantaneous	[9]
	carbon nanoparticles	1.25 V	0.1 μA	0.125 μW	constant	[46]
	Al_2_O_3_	4 V	18 μA	72 μW	instantaneous	[49]
	MoS_2_	0.019 V	6.24 μA	0.11856 μW	constant	[50]
	Ni–Al LDH	0.6 V	0.3 μA	0.18 μW	constant	[52]
	TiO_2_	0.5 V	8 μA	4 μW	constant	[54]
	Ti_3_C_2_T*_x_* MXene nanosheets	0.69 V	7.55 mA	5.2095 mW	instantaneous	[55]
	graphene oxide	0.3 V	3 mA	0.9 mW	instantaneous	[61]
	graphene oxide	1.2 V	136 nA	163.2 nW	instantaneous	[64]
	carbon nanoparticle film	0.6 V	0.3 μA	0.18 μW	instantaneous	[89]
organic nanomaterials	PVP	6 V	70 μA	420 μW	instantaneous	[75]
	P4VP	3 V	1.5 μA	4.5 μW	instantaneous	[78]
	FEP	—	3 μA	—	instantaneous	[21]
	biological nanofibers (NFs)	0.115 V	200 nA	23 nW	constant	[85]
	porous polydopamine (g-PDA)	0.52 V	3 mA	1.56 mW	instantaneous	[84]
	protein nanowires	0.5 V	200 nA	100 nW	constant	[86]
	gelatin molecules	0.71 V	8 μA	5.5 μW	instantaneous	[87]
	paper	0.25 V	12 nA	3 nW	constant	[79]
	cellulose nanofibrils	0.1 V	15 nA	1.5 nW	constant	[80]
	natural wood	0.3 V	10 μA	3 μW	constant	[81]
	CNF/GO composite films	0.286 V	—	—	constant	[83]
	ionic liquid (Omim^+^ Cl^−^)	0.2 V	50 μA	10 μW	constant	[88]

### 4 Applications of MEG technology

**Figure 10:**
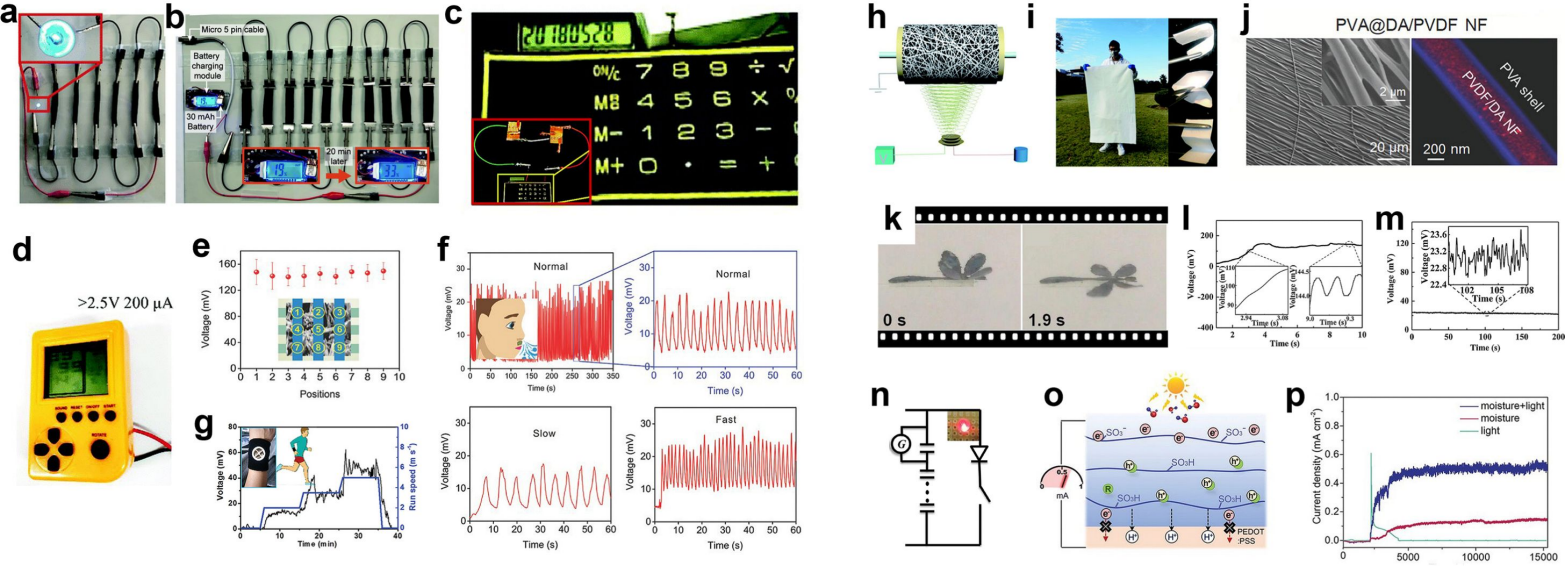
(a) MEGs power directly a blue LED. (b) MEGs charge a battery. Figure 10a,b were reproduced from [55] (“Towards Watt-scale hydroelectric energy harvesting by Ti_3_C_2_T*_x_*-based transpiration-driven electrokinetic power generators“, © 2022 J. Bae et al., published by Royal Society of Chemistry, distributed under the terms of the Creative Commons Attribution NonCommercial 3.0 Unported License, https://creativecommons.org/licenses/by-nc/3.0/). This content is not subject to CC BY 4.0. (c) MEG power an eight-digit calculator. (d) MEG light up the LCD screen of a handheld game console. Figure 10c and 10d were republished with permission of The Royal Society of Chemistry from [90] (“An efficient polymer moist-electric generator” by Xu, Tong et al., Energy Environ. Sci., vol. 12, issue 3, © 2019); permission conveyed through Copyright Clearance Center, Inc. This content is not subject to CC BY 4.0. (e) Touch sensor. (f) Breath detection sensor, different breathing modes yield different output voltages. Figure 10e,f were reproduced from [54], Shen, D. et al., “Self-Powered Wearable Electronics Based on Moisture Enabled Electricity Generation”, Adv. Mater., with permission from John Wiley and Sons. Copyright © 2018 WILEY-VCH Verlag GmbH & Co. KGaA, Weinheim. This content is not subject to CC BY 4.0. (g) Motion monitoring sensor. Figure 10g was reproduced from [80], Li, Mingjie et al., “Biological Nanofibrous Generator for Electricity Harvest from Moist Air Flow”, Adv. Funct. Mater., with permission from John Wiley and Sons. Copyright © 2019 WILEY-VCH Verlag GmbH & Co. KGaA, Weinheim. This content is not subject to CC BY 4.0. (h) Schematic diagram of the electrospinning apparatus and (i) large-area, flexible, and deformable nanofiber fabric for MEGs. Figure 10h and 10i were republished with permission of The Royal Society of Chemistry from [77] (“Electrospun nanofiber fabric: an efficient, breathable and wearable moist-electric generator” by Sun, Zhaoyang et al., Journal of Materials Chemistry A., vol. 9, issue 11, © 2021); permission conveyed through Copyright Clearance Center, Inc. This content is not subject to CC BY 4.0. (j) SEM image showing a highly aligned PVA@DA/PVDF NF array. The LSCM image shows PVA-wrapped DA/PVDF NFs. Figure 10j was reproduced from [76], Li, T. et al., “Power Generation from Moisture Fluctuations Using Polyvinyl Alcohol-Wrapped Dopamine/Polyvinylidene Difluoride Nanofibers”, Small, with permission from John Wiley and Sons. Copyright © 2021 WILEY-VCH GmbH. This content is not subject to CC BY 4.0. (k) MEGs in micro/nano drives. (l, m) The electrical output signal generated by the generator during humidification and dehumidification processes. (n) The equivalent circuit diagram of the PEDOT:PSS/PVDF double-layer generator charging a capacitor to power a LED. Figure 10k–n were reprinted from [92]. This article was published in Sensors and Actuators B: Chemical, vol. 255, by G. Wang; H. Xia; X.-C. Sun; C. Lv; S.-X. Li; B. Han; Q. Guo; Q. Shi; Y.-S. Wang; H.-B. Sun, “Actuator and generator based on moisture-responsive PEDOT: PSS/PVDF composite film“, pages 1415-1421, Copyright Elsevier (2018). This content is not subject to CC BY 4.0. (o) Schematic diagram of a light-coordinated MEG and (p) output power variation curve. Figure 10o and 10p were reproduced from [93], Bai, J. et al., “Sunlight-Coordinated High-Performance Moisture Power in Natural Conditions”, Adv. Mater., with permission from John Wiley and Sons. Copyright © 2022 WILEY-VCH GmbH. This content is not subject to CC BY 4.0.

As a renewable green energy source, MEGs have great application potential, and devices based on humidity-responsive self-powering have been applied in many fields. In terms of direct power supply applications, current MEGs can directly power low-power electronic devices such as LCDs and LEDs, or charge capacitors and batteries to drive higher-power devices (Figure 10a–d) [55,90]. Because MEG devices can be rapidly mass-fabricated by methods such as stencil printing [91], MEGs should become a strong candidate for green energy in the near future.

MEGs are also widely used in sensors [54]. For example, a moisture-eletric touch sensor array can provide uniform and sensitive touch feedback (Figure 10e). As shown in Figure 10f, a breath detector can monitor different breathing patterns, including short breaths, normal breaths, and deep breaths. The sweat detector delivers voltage signals a function of the amount of sweat when the human body is exercising (Figure 10g).

A novel and promising application of MEGs has also been demonstrated recently, namely, the use of MEG materials to prepare textile fibers [76–77]. This power-generating textile fiber offers new horizons as they can harvest energy anytime and anywhere. For example, in Sun’s work, a breathable, flexible, and deformable large-faced textile was demonstrated, which utilizes polyethylene oxide (PEO) to achieve an output of up to 0.83 V (Figure 10i). There are also PVA@DA/PVDF NF composites that can efficiently generate instantaneous voltages up to 0.5 V from ambient moisture (Figure 10j). However, because of different issues regarding daily practical applications, such as sweat corrosion, biological toxicity on human skin, and the need for suitable electrode materials to collect the electric energy, wearable MEG textiles still need require further investigation.

MEGs also have potential in other research fields. In micro/nano-driven devices [92], a hygro-responsive layer can use ambient humidity to provide a continuous power supply for the device (Figure 10k–n). When being combined with optoelectronic devices, photo-excited carriers and moisture-induced charge separation promote each other. The open-circuit voltage of the MEG from the photoelectric effect is increased by more than 10%, and the short-circuit current is increased by 300% (Figure 10p) [93]. A variety of power generation mechanisms to improve the output power have practical value, and the self-powered bias layer of MEGs may contribute to improving the output power in the future.

## Conclusion

MEGs are based on the direct utilization of fluid potential energy in nanoarchitectonics and are renewable energy source. The advantage of MEG devices is that the devices can convert moisture or water energy from the environment directly into electrical energy. For a theoretical understanding of MEGs, the historical lineage of MEGs has been reviewed above, and its principles have been traced back to their origin. Subsequently, this review provides a comprehensive coverage of articles in the emerging field of MEGs. Materials are divided into organic and inorganic materials, and the link between the fields of nanoarchitectonics and MEGs is introduced. Parameters that have an effect on MEGs were summarized. These parameters include material type, nanoarchitectonics, functional group content, humidity, fluid ion type, fluid ion concentration, fluid velocity, and carrier transport structure. In terms of electrical output, the output power of the current MEGs is still very low. As shown in Table 1, the existing MEG research roughly has two power output modes, instantaneous output and constant output. Currently, instantaneous-output devices can provide up a maximum electrical output of 0.69 V and 7.55 mA. Devices with constant output can reach an electrical output of 0.5 V and 8 μA. Instantaneous-output devices provide high open-circuit voltage, which can reach 4 V or more, but because of the low frequency, the total energy that can be provided is limited. In constant-output devices, the open-circuit voltages of most devices are less than 1 V, and most short-circuit currents do not exceed 10^−4^ A, so power output is limited to less than 0.1 mW·cm^−2^. Existing MEG device designs are still in the preliminary stage, and various emerging designs are being examined in MEG applications, such as porous material devices, moisture gradient designs, ion concentration difference devices, and ionic liquid devices. All need more experiments to prove and explore the underlying concepts. In future MEG research, with further exploration of different parameters and structural designs, the output power is expected to increase with updated designs. Most important is to further explore the interaction mechanisms of solids and liquids at the nanoscale to fundamentally investigate the possibility of increasing the output power of MEGs.

MEGs have great potential for applications as power generators for wearable self-powered pressure sensors, respiratory monitors, motion detectors, power sources for small electronic devices (LEDs, LCD screens, and electronic watches), power-generating textiles, and self-powered layers for other micro- and nanoscale devices. There are already a lot of references available for these applications. MEGs can greatly reduce the weight and increase the portability compared with traditional chemical batteries. Therefore, the application and development of MEGs in the future should focus on miniaturization and increased power output. Also, MEGs can be used in a similar way to solar cells but with fewer limitations. It is probably not too long before MEGs will be successfully applied in practical applications and provide green energy for more electronic devices.
